# A Conversation with the Grand Lama

**Published:** 1889-04

**Authors:** 


### A CONVERSATION WITH THE GRAND LAMA.

We quote the following from George Kennan's description of his visit to the Grand Lama of the Trans-Baikal, in the March Century.

"After dinner I had a long talk with the Grand Lama about my native country, geography, and the shape of the earth. It seemed very strange to find anywhere on the globe, in the nineteenth century, an educated man and high ecclesiastical dignitary who had never even heard of America, and who did not feel at all sure that the world is round. The Grand Lama was such a man.

'You have been in many countries,' he said to me through the interpreter, 'and have talked with the wise men of the West ; what is your opinion with regard to the shape of the earth ?'

'I think,' I replied, 'that it is shaped like a great ball.'

'I have heard so before,' said the Grand Lama, looking thoughtfully away into vacancy. 'The Russian officers whom I have met have told me that the world is round. Such a belief is contrary to the teachings of our old Thibetan books, but I have observed that the Russian wise men predict eclipses accurately ; and if they can tell beforehand when the sun and the moon are to be darkened, they probably know something about the shape of the earth. Why do you think that the earth is round ?'

'I have many reasons for thinking so,' I answered ; 'but perhaps the best and strongest reason is that I have been around it.'

This statement seemed to give the Grand Lama a sort of mental shock.

'How have you been around it ?' he inquired. 'What do you mean by "round it ?" How do you know that you have been around it ?'

'I turned my back upon my home,' I replied, 'and traveled many months in the course taken by the sun. I crossed wide continents and great oceans. Every night the sun set before my face and every morning it rose behind my back. The earth always seemed flat, but I could not find anywhere an end or an edge ; and at last, when I had traveled more than thirty thousand versts, I found myself again in my own country and returned to my home from a direction exactly opposite to that which I

had taken in leaving it. If the world was flat, do you think I could have done this?'

'It is very strange,' said the Grand Lama, after a thoughtful pause of a moment. 'Where is your country? How far is it beyond St. Petersburg?'

'My country is farther from St. Petersburg than St. Petersburg is from here,' I replied. It lies almost exactly under our feet?' and if we go directly through the earth, that would be the shortest way to reach it.

'Are your countrymen walking around there heads downward under our feet? asked the Grand Lama with evident interest and surprise, but without any perceptible change in his habitually impassive face.

'Yes,' I replied; 'and to them we seem to be sitting heads downward here.'

'The Grand Lama then asked me to describe minutely the route that we had followed in coming from America to Siberia, and to name the countries through which we had passed. He knew that Germany adjoined Russia on the west, he had heard of British India and of England—probably through Thibet,—and he had a vague idea of the extent and situation of the Pacific Ocean; but of the Atlantic and of the continent that lies between the two great oceans, he knew nothing.

After a long talk, in the course of which we discussed the sphericity of the earth from every possible point of view, the Grand Lama seemed to be partly or wholly convinced of the truth of that doctrine, and said, with a sigh, 'it is not in accordance with the teachings of our books; but the Russians must be right.'

It is a somewhat remarkable fact that Dr. Erman, the only foreigner who had seen the lamasery of Goose Lake previous to our visit, had an almost precisely similar conversation concerning the shape of the earth with the man who was then (in 1828) Grand Lama. Almost sixty years elapsed between Dr. Erman's visit and ours, but the doctrine of the sphericity of the earth continued throughout that period to trouble ecclesiastical minds in this remote East-Siberian lamasery; and it is not improbable that sixty years hence some traveler from the western world may be asked by some future Grand Lama to give his reasons for believing the world to be a sphere."

---

An eminent Physician says anyone can be cured of the Tobacco appetite, by chewing Green Plantain leaves and swallowing the juice. It destroys the desire entirely, so much so, that after chewing these leaves awhile, if they wished to return to the habit, had to learn to love it over again. Every person that has tried it, finds this to be true. Plantain grows in our back yards and other neglected places.

